# First-in-human study of CPL’116 – a dual JAK/ROCK inhibitor – in healthy subjects

**DOI:** 10.3389/fphar.2025.1583723

**Published:** 2025-04-01

**Authors:** Piotr J. Rudzki, Katarzyna Jarus-Dziedzic, Dorota Włodarczyk, Michał Kaza, Piotr Pankiewicz, Agnieszka Gierczak-Pachulska, Martyna Banach, Beata Zygmunt, Cezary Piwowarczyk, Paweł Żero, Daniel Rabczenko, Agnieszka Segiet-Święcicka, Maciej Wieczorek

**Affiliations:** ^1^ R&D Center, Celon Pharma S.A., Kazuń Nowy, Poland; ^2^ Clinical Site, BioResearch Group, Kajetany, Poland; ^3^ CleanDataLabs, Warsaw, Poland

**Keywords:** phase I (drug development), safety, pharmacokinetics, pharmacodynamics, CPL’116 (previously CPL409116), Janus kinase (JAK), Rho kinase (ROCK), inflammation

## Abstract

**Background:**

CPL’116 is a novel Janus kinase (JAK) and Rho-associated coiled-coil containing protein kinase (ROCK) dual inhibitor and a promising drug candidate for the treatment of inflammatory and fibrotic diseases. We conducted this first-in-human, Phase I clinical trial to evaluate the safety, pharmacokinetics (PK), and exploratory pharmacodynamics (PD) of CPL’116 in healthy subjects.

**Methods:**

Phase I clinical trial in healthy White volunteers was conducted after single (n = 21, 10–300 mg) and multiple (n = 32, 30–240 mg or placebo, 14-day b.i.d.) administrations of CPL’116 including a food effect study (n = 12, 120 mg). The multiple ascending dose part was double-blinded and placebo-controlled. The primary endpoint was safety evaluation, and the secondary endpoint was PK. Exploratory PD was studied by measuring the inhibition of JAK and ROCK in the blood by assessing STAT1, STAT5, and MLC phosphorylation.

**Results:**

Safety parameters were comparable between the placebo and active treatment groups, with no clinically meaningful variations in the safety parameters between the cohorts. No deaths or serious adverse events (SAEs) were reported. No influence on hematological parameters (neutrophil count, red cell distribution width, and mean corpuscular volume) was observed. Plasma C_max_ and AUC increased proportionally in the dosing range of 60–240 mg. Median t_max_ ranged 2–3 h. Food increased the absorption of CPL’116. Compared to placebo, CPL’116 at 240 mg dose showed a decrease in the phosphorylation of STAT1 (Days 1 and 14, p < 0.05) and STAT5 (Day 14, p < 0.05). A decrease in MLC phosphorylation indicated a potential trend at p < 0.1.

**Conclusion:**

CPL’116 was safe and well-tolerated by healthy subjects. The PK profile is well suited for twice-daily administration and justifies further clinical development. Exploratory PD studies indicated the ability of CPL’116 to affect the JAK and ROCK pathways in humans, hinting at its potential therapeutic role in diseases benefiting from its dual mode of action. The positive results of this study indicate the possibility of developing a novel class of therapeutics that address both inflammatory and fibrotic processes.

**Clinical Trial RegistrationMethods:**

clinicaltrials.gov, identifier NCT04670757.

## 1 Introduction

Immune-mediated inflammatory diseases (IMIDs) encompass a wide range of heterogeneous disorders including asthma, inflammatory bowel disease, multiple sclerosis, rheumatoid arthritis, psoriasis, and atopic dermatitis. The increasing prevalence of IMIDs is constantly driven by the growth and aging of the population, as well as the interplay of genetic and environmental factors (Wu D et al., 2023). As it has become a global challenge for health systems, there is an urgent need for safe and effective therapies that will not only temporarily relieve symptoms, but also stop the progression of IMIDs and further health complications that often accompany them.

Janus kinase (JAK) is a family of non-receptor protein-tyrosine kinases, including JAK1, JAK2, JAK3, and tyrosine kinase 2 (TYK2). JAKs play a central role in transmitting signals from growth factors and type I and II cytokine receptors (Ghoreschi, Laurence, and O’Shea, 2009; O’Shea et al., 2015). Upon binding to specific receptors, JAKs undergo phosphorylation, leading to Signal Transducer and Activator of Transcription (STAT) activation, which drives the expression of pro-inflammatory genes involved in chronic inflammation in autoimmune diseases (O’Shea et al., 2015). Several small-molecule JAK inhibitors have already been integrated into the clinical practice. Among them, upadacitinib, tofacitinib, peficitinib, filgotinib, delgocitinib, and abrocitinib have been successfully used to treat inflammatory diseases, such as rheumatoid arthritis, psoriatic arthritis, atopic dermatitis, and ulcerative colitis. Clinical trials of JAK inhibitors in selected IMIDs have shown comparable or superior results to those achieved with biological agents (O’Shea et al., 2015; Nash et al., 2021; Cai et al., 2024), indicating that they are an attractive class of modern therapeutic agents (McInnes and Gravallese, 2021). Despite their remarkable therapeutic benefits, JAK inhibitors raise some safety concerns that restrict their use (Song et al., 2022; Zhang et al., 2023; Ma et al., 2024). Based on the ORAL Surveillance study findings (Winthrop and Cohen, 2022), both the FDA and EMA issued box warnings regarding the increased risk of serious cardiovascular events, cancer, blood clots, and mortality associated with these drugs. Following the analysis of data from the tofacitinib safety trial, the FDA concluded that there is an increased risk of serious events such as heart attack or stroke, cancer, blood clots, and death. Based on the same mechanism of action, the FDA has considered similar risks for baricitinib and upadacitinib. The EMA recommended that abrocitinib, filgotinib, baricitinib, upadacitinib, and tofacitinib should only be used in the following populations if no suitable treatment options exist: in patients aged 65 years or above, those at increased risk of heart attack, stroke, or cancer, and current or previous long-term smokers. EMA also recommended the use of JAK inhibitors with caution in patients at risk of blood clotting in the lungs and deep veins.

The Rho-associated coiled-coil containing protein kinase (ROCK) family comprises two highly homologous serine-threonine kinases, ROCK1 and ROCK2, which are key downstream effectors of the small GTPase RhoA. ROCK is involved in numerous biological processes, such as cell migration, adhesion, proliferation, differentiation, and apoptosis signaling (Amin et al., 2013; Amano, Nakayama, and Kaibuchi, 2010). A key aspect of these pathways is the upregulation of transforming growth factor β (TGF-β), which is a signal for increased fibroblast differentiation observed in the pathogenesis of idiopathic pulmonary fibrosis (IPF) and interstitial lung disease in the course of rheumatoid arthritis (ILD-RA) (Julian and Olson, 2014; Yang et al., 2012; Zhou et al., 2013). In addition, ROCK and myosin light chain (MLC) protein-mediated signaling contributes to stress fiber assembly and actomyosin contraction, affecting cardiovascular disease development (Hartmann, Ridley, and Lutz, 2015). Finally, studies have shown that ROCK inhibitors, by suppressing NF-κB activation and therefore reducing the production of tumor necrosis factor α (TNF-α) and interleukin 1β (IL-1β), exhibit anti-inflammatory effects in various pathological conditions, including acute lung injury and rheumatoid arthritis (Tasaka et al., 2005; He et al., 2008). Pharmacological inhibition of ROCK has been also found to lower IL-17 and IL-21 levels, both of which play a role in autoimmune disease progression (Rozo et al., 2017). Four small-molecule ROCK inhibitors (fasudil, ripasudil, netarsudil, and belumosudil) have been approved for clinical use with indications for cerebral vasospasm, open-angle glaucoma, ocular hypertension, and chronic graft-versus-host disease. However, their great potential as promising therapies for inflammatory and fibrotic diseases remains underexplored in practice.

Given this knowledge, the simultaneous targeting of both the JAK and ROCK pathways represents a potentially novel therapeutic approach for diseases characterized by both inflammatory and fibrotic processes such as idiopathic pulmonary fibrosis (Nash et al., 2021) or renal fibrosis (Baba et al., 2015; Wang et al., 2022). Rheumatoid arthritis presents itself as a particularly compelling therapeutic target, considering the high incidence of cardiovascular complications in the RA patient population (Johri et al., 2023), as well as the development of interstitial lung disease during the course of the disease (McInnes and Gravallese, 2021). We believe that the well-established anti-inflammatory effects of JAK inhibitors could be further potentiated by the cardioprotective and anti-fibrotic properties of ROCK inhibitors. In this context, CPL’116 (previously CPL409116, CAS No. 2250013-34-2), a novel dual inhibitor targeting JAK (more specific to JAK1 and JAK3 than JAK2) and ROCK, has great therapeutic potential. Its high potency against target kinases was confirmed in preclinical studies (Dulak-Lis et al., 2021). The mechanism of action has been validated in various studies, confirming the involvement of STAT1/5 and MLC pathways. In lupus-prone mice, CPL’116 showed effectiveness in preventing nephropathy and improving renal function (Dulak-Lis et al., 2021). It also exhibited potential in preclinical models of diseases, such as psoriasis and arthritis (unpublished data). Additionally, the feasibility of pulmonary delivery of CPL’116 was evaluated *in vitro* (Rzewińska et al., 2024).

Here, we present the results of a first-in-human clinical study evaluating the safety, pharmacokinetics, and pharmacodynamic properties of CPL’116 after single and multiple administrations in healthy participants (volunteers). The effect of food intake on the bioavailability of CPL’116 was also investigated.

## 2 Methods

### 2.1 Study design and participants

This single-center Phase I study consisted of three separate parts:

• Part A–a single ascending dose (SAD)
• Part A additional–food effect
• Part B–multiple ascending dose (MAD).

In each part, participants remained hospitalized for at least 12 h before the first CPL’116 administration to 24 h after the last dose in the SAD and MAD parts and each dose in the food effect part. During hospitalization, standardization of diet, proper fluid administration, and daily activity were ensured by clinical site personnel.

The SAD part of the trial was a conventional 3 + 3 design study consisting of seven cohorts, each with three participants. Based on data from preclinical and toxicological studies, the calculated starting dose was 10 mg CPL’116. Doses were escalated until (1) the occurrence of dose-limiting toxicities (DLT), defined as grade 3 toxicity adverse events and determination of maximum tolerated dose (MTD), or (2) reaching the highest planned dose. As an additional safety measure, a sentinel dosing approach was applied to detect acute safety risks during new dose administration: the interval between participants in the cohort was at least 24 h, whereas the interval between the first participant in subsequent cohorts was at least 7 days. The following single doses of CPL’116 were studied under fasting conditions: 10, 30, 60, 120, 180, 240, and 300 mg. Follow-up included an on-site visit on Day 7 (to perform blood chemistry, complete blood count, urinalysis test, and blood pregnancy test for females), a telephone call regarding any adverse events and health condition on Day 14, and an on-site visit on Day 32 (+2 days) for females to perform a urine pregnancy test.

In the subsequent part of the study, the effect of food on CPL’116 bioavailability was evaluated in 12 participants during two periods, separated by a 7-day washout period. In period I, a single 120 mg dose of CPL’116 was administered under fasting conditions, whereas in period II, the same dose was administered after a standardized high-fat breakfast. The follow-up was the same as that in the SAD part.

The MAD part was a double-blind, placebo-controlled study with four cohorts consisting of eight participants each receiving CPL’116 or placebo in a 3:1 ratio. Based on the safety and pharmacokinetic outcomes from the SAD part of the study, CPL’116 at doses of 30, 60, 120, and 240 mg or placebo was administered twice daily (every 12 h) on an empty stomach for 14 consecutive days. Follow-up was similar to the SAD part, with the same timing related to the last CPL’116 administration: on-site visit was on Day 21, telephone call was on Day 28, and on-site visit was on Day 45 (+2 days) for females.

This study was conducted on healthy males and females. Prior to enrolment, written informed consent was obtained from each participant. A total of 65 participants (21 in the SAD part, 12 in the food effect part, and 32 in the MAD part) were enrolled. The study group included White participants aged 18–55 years, with a body mass index (BMI) ranging from 18.5 to 29.9 kg/m^2^ who successfully completed medical screening. The participants were in good health, as assessed by their routine medical history, physical examination, vital signs, and laboratory data. They were non-smokers and had not used tobacco products for at least 3 months prior to the study. Individuals who had recently donated blood, participated in another trial within 3 months before the start of this study, received prescription or over-the-counter medications during the trial, or were pregnant or breastfeeding were excluded. The participants practiced effective contraception throughout the study period. The participants could only participate in one part of the study.

The study was conducted at the facilities of the BioResearch Group Ltd. (Kajetany, Poland) between 8 December 2020, and 8 September 2021, in accordance with Good Clinical Practice (GCP), respective ICH guidelines, and principles of the Declaration of Helsinki and its amendments.

### 2.2 Study product administration

The immediate-release tablets were designed to fit the dosing scheme in the study and contained 10 or 60 mg of CPL’116. The main challenge in the development of pharmaceutical formulation was the limited solubility of the active pharmaceutical ingredient. This issue was resolved by applying hot-melt extrusion to increase the bioavailability of CPL’116. The study products, including a placebo formulated as tablets identical to the active product, were manufactured by Celon Pharma S.A. Impurity profile was described previously (Gurba-Bryśkiewicz et al., 2022).

The study products were orally administered with a glass of water (250 mL). After product administration, the participant’s oral cavity was checked by an investigator using a laryngological spatula and medical flashlight.

### 2.3 Safety assessment

The primary endpoint of this study was the safety evaluation of CPL’116 following oral administration for both single and multiple 14-day dosing regimens. The assessment encompassed an examination of adverse events, including a comparative analysis of pre- and post-dose parameters of heart rate, blood pressure, and hematological, biochemical, and urinalysis results. In the MAD part, the changes in these parameters from baseline were compared between the active and placebo groups.

### 2.4 Pharmacokinetic analysis

The secondary objectives were the evaluation of pharmacokinetics and the influence of food on CPL’116 bioavailability. Blood samples for PK analysis were collected via cannula in sodium citrate tubes. During the SAD and food effect parts, 4.5 mL of blood was collected at 0 (pre-dose) and 0.25, 0.5, 0.75, 1, 2, 3, 4, 5, 6, 7, 8, 10, 12, 24, and 48 h after CPL’116 administration. The following samples were collected during the MAD part: 13 samples on Days 1, 8, and 14 from 0 (pre-dose) to 12 h post-dose; on Days 2–7 and 9–13 morning pre-dose and 2 h post-dose; and on Day 15 samples 24 h and 36 h (after the last administration on Day 14). CPL’116 and its M3 metabolite concentrations in the plasma were measured in compliance with Good Laboratory Practice. Isotope-labelled internal standards were used, and the HPLC/MS/MS bioanalytical method (see Supplementary Material) was validated according to EMA (European Medicines Agency, 2011) and FDA (U.S. Food and Drug Administration, 2018) guidelines.

Pharmacokinetic parameters were computed individually for each subject using a non-compartmental modelling approach and actual sampling times. The C_max_ and t_max_ were determined directly from the plasma concentration *versus* time data. The terminal phase elimination rate constant (K_el_) was estimated using log-linear regression of at least three last concentrations. The t_1/2_ value was calculated as 0.693/K_el_. The AUCs were calculated using the linear trapezoidal rule. The following parameters were evaluated for CPL’116:

• In the SAD and food effect parts: C_max_, AUC_0–24h_, t_max_, t_1/2_
• in the MAD part: C_max_, AUC_0–12h_, t_max_, t_1/2_, and accumulation ratio (Day 8 vs Day 1 and Day 14 vs Day 1).

Additionally, the individual ratios of C_max_, AUCs, and differences in t_max_ between CPL’116 and its M3 metabolite were calculated.

### 2.5 Exploratory pharmacodynamics

The inhibition of JAK and ROCK in the blood was assessed by phosphorylation of STAT1 and STAT5 – JAK molecular target, employed previously for tofacitinib (Dowty et al., 2014) – as well as MLC–ROCK molecular target. During the MAD part, approximately 4 mL of blood was collected for pharmacodynamic assessments in K_2_EDTA tubes at pre-dose (Day 1), 2 h after the second administration (Day 1), and 2 h after the last administration (Day 14). The influence of CPL’116 on the phosphorylation status of signaling molecules was determined by comparing the fluorescence intensity in blood samples collected before and after CPL’116 administration, as measured by fluorescence-activated cell sorting (see Supplementary Material).

### 2.6 Statistical analysis

Statistical analysis was performed by Instytut Edukacji (currently CleanDataLabs), Poland. No formal sample size determination was performed for dose escalation, because the sample sizes were not based on statistical power. In each part of the study, randomization numbers were assigned according to the participants’ arrival at the clinical site on Day 0. The SAD and food effect were the open-label parts of the study. The MAD part was double blind. The Sponsor provided a randomization table for the clinical site in sealed envelopes. Two clinical staff members were unblinded to this study: a pharmacist and a quality control person responsible for verifying the proper administration of the products. The bioanalytical laboratory was blinded to the randomization table until all concentrations were measured. The study blind was lifted after the clinical phase concluded, allowing for statistical analysis.

Descriptive methods (without formal hypothesis testing) were used to analyze the data. The analysis of adverse events involved the use of mixed models that incorporated fixed effects for time and dose along with a random effect accounting for individual subjects.

The effect of food on CPL’116 bioavailability was assessed using 90% confidence intervals of the geometric mean ratios (fed vs fasting conditions) calculated based on the ANOVA models for log-transformed C_max_, AUC_0-t_, AUC_0-inf_, t_1/2_, and K_el_ with the conditions + subject fixed effects. Owing to the exploratory nature of this analysis, no acceptable range was set. The non-parametric Wilcoxon test was used to determine the difference in t_max_ between fed and fasting conditions. Statistical significance was set at p < 0.05. The proportionality/linearity of the relationship between pharmacokinetic parameters and CPL’116 dose was assessed by: (1) ANOVA and 90% confidence intervals of log-transformed dose-normalized parameters, and (2) estimation of the mean slope (and 90% confidence intervals) of the log-transformed parameters against log-dose.

Exploratory PD analysis was conducted using two-way ANOVA for repeated measures, followed by *post hoc* Šidák multiple comparison tests and Spearman correlation coefficients with 95% confidence intervals (CIs). Furthermore, 95% CIs were reported for placebo-normalized PD parameters and a two-sided one-sample t-test was used to assess the arithmetic mean against a theoretical mean of 1. The significance level was set at α = 0.05.

## 3 Results

### 3.1 Demographics and baseline characteristics

**FIGURE 1:**
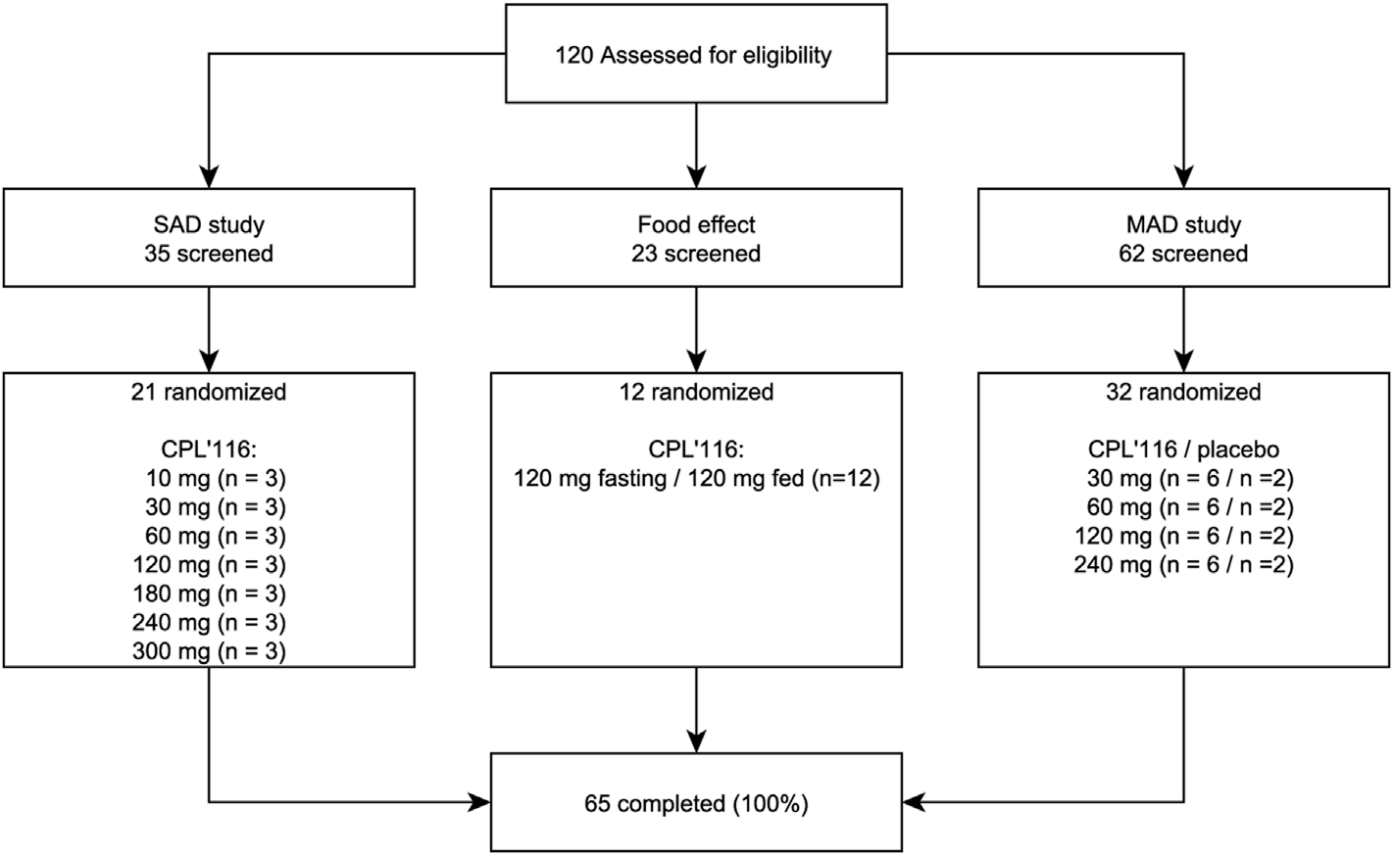
Subject disposition in the first-in-human study of CPL’116.

A total of 120 White subjects were screened and 65 were enrolled in the study: 21 participated in the SAD part, 12 in the food effect part, and 32 in the MAD part (Figure 1). Females constituted 54% of the study population (Table 1). All participants completed the study.

**TABLE 1:** Baseline demographics

Characteristic	SAD (n=21)	Food effect (n=12)	MAD CPL’116 (n = 24)	MAD Placebo (n = 8)
Age, years, mean ± SD	40.1 ± 8.8	30.1 ± 8.4	35.6 ± 8.8	35.3 ± 11.2
Sex, n (%)				
Female	15 (71%)	6 (50%)	11 (46%)	3 (38%)
Male	6 (29%)	6 (50%)	13 (54%)	5 (62%)
Ethnicity, n (%)				
White	21 (100%)	12 (100%)	24 (100%)	8 (100%)
Weight, kg, mean ± SD	73.6 ± 8.9	71.2 ± 13.4	77.6 ± 12.3	76.6 ± 12.1
BMI, kg/m^2^, mean ± SD	25.5 ± 2.9	23.9 ± 2.8	25.3 ± 2.3	25.0 ± 2.3

BMI, body mass index; MAD, multiple ascending dose; SAD, single ascending dose; SD, standard deviation.

### 3.2 Safety

CPL’116 demonstrated a good safety profile following both single and multiple administrations of all the tested doses. No DLTs were observed up to the highest planned dose in this study; therefore, the MTD for CPL’116 was not determined. All reported adverse events (AEs) were classified as non-serious, ranging from mild to moderate (Table 2). No clinically meaningful variations in safety parameters were detected between the cohorts after a single administration and no food effect on safety outcomes was identified. Furthermore, no discernible alterations in safety parameters were observed over the 2-week administration period, and no evident dose-dependent trends were observed. The safety profiles of the placebo and active treatment groups remained comparable.

**TABLE 2:** Summary of safety data and adverse events reported by two or more subjects dosed with CPL’116 or placebo.

CPL’116 dose	SAD	Food effect part	MAD					TOTAL CPL’116
	10-300 mg	120 mg	30 mg	60 mg	120 mg	240 mg	Placebo	
subjects in cohort/s	n = 21	n = 12	n = 6	n = 6	n = 6	n = 6	n = 8	n = 57
Number of AEs reported (% subjects with AE)	3 (14%)	8 (42%)	3 (50%)	6 (50%)	8 (50%)	20 (100%)	6 (50%)	48 (47%)
mild (% AEs)	3 (100%)	4 (50%)	0 (0%)	2 (33%)	5 (62%)	12 (60%)	5 (83%)	22(46%)
moderate (% AEs)	0 (0%)	4 (50%)	3 (100%)	4 (6%)	3 (38%)	8 (40%)	1 (17%)	26 (54%)
severe (% AEs)	0 (0%)	0 (0%)	0 (0%)	0 (0%)	0 (0%)	0 (0%)	0 (0%)	0 (0%)
Serious AEs (%)	0 (0%)	0 (0%)	0 (0%)	0 (0%)	0 (0%)	0 (0%)	0 (0%)	0 (0%)
Treatment-related AEs (%)	0 (0%)	0 (0%)	0 (0%)	0 (0%)	0 (0%)	0 (0%)	2 (33%)^a^	0 (0%)
AEs leading to study drug discontinuation (% AEs)	0 (0%)	0 (0%)	0 (0%)	0 (0%)	0 (0%)	0 (0%)	0 (0%)	0 (0%)
Deaths (%)	0 (0%)	0 (0%)	0 (0%)	0 (0%)	0 (0%)	0 (0%)	0 (0%)	0 (0%)
AEs reported in n ≥ 2 subjects	Number of AEs (% subjects with AE)							
headache	1 (8%)	0 (0%)	1 (17%)	0 (0%)	2 (33%)	4 (67%)	2 (25%)	8 (14%)
hematoma	0 (0%)	0 (0%)	3 (50%)	0 (0%)	0 (0%)	0 (0%)	0 (0%)	3 (5%)
bruise	0 (0%)	0 (0%)	0 (0%)	0 (0%)	1 (17%)	2 (33%)	0 (0%)	3 (5%)
increased leucocytes level	0 (0%)	2 (17%)	0 (0%)	0 (0%)	1 (17%)	0 (0%)	1 (12.5)	3 (5%)
increased bilirubin level	2 (10%)	0 (0%)	0 (0%)	0 (0%)	0 (0%)	1 (17%)	0 (0%)	3 (5%)
diarrhea	0 (0%)	0 (0%)	0 (0%)	0 (0%)	0 (0%)	2 (33%)	0 (0%)	2 (4%)
excessing sweating	0 (0%)	0 (0%)	0 (0%)	0 (0%)	0 (0%)	1 (17%)	1 (13%)	1 (2%)
increased ALT level	0 (0%)	0 (0%)	0 (0%)	0 (0%)	0 (0%)	2 (33%)	0 (0%)	2 (4%)
decreased aPTT level	0 (0%)	0 (0%)	0 (0%)	0 (0%)	2 (33%)	0 (0%)	0 (0%)	2 (4%)
nausea	0 (0%)	0 (0%)	0 (0%)	0 (0%)	0 (0%)	2 (33%)	0 (0%)	2 (4%)

^a^ AEs classified as possibly related to the study drug;

AE, adverse event; ALT, alanine aminotransferase; aPTT, activated partial thromboplastin time; MAD, multiple ascending dose; SAD, single ascending dose.

After single dosing, AEs were reported in 3 of 21 participants (14%) in the SAD part, and in 5 of 12 participants (42%) in the food effect part, accounting for a total of 11 AEs. The most frequently reported AEs included elevated total bilirubin levels (SAD part, n = 2), increased leukocyte counts, and elevated creatine kinase serum levels (food effect part, n = 2 each). No significant relationship between CPL’116 dose and the odds of any AE occurrence was identified [OR 0.997 (95% CI 0.982–1.009); p = 0.658]. No significant relationship between fed/fasting conditions and the odds of any AE occurrence was identified in the food effect part [OR 2.829 (95% CI 0.383–49.670); p = 0.344].

**FIGURE 2:**
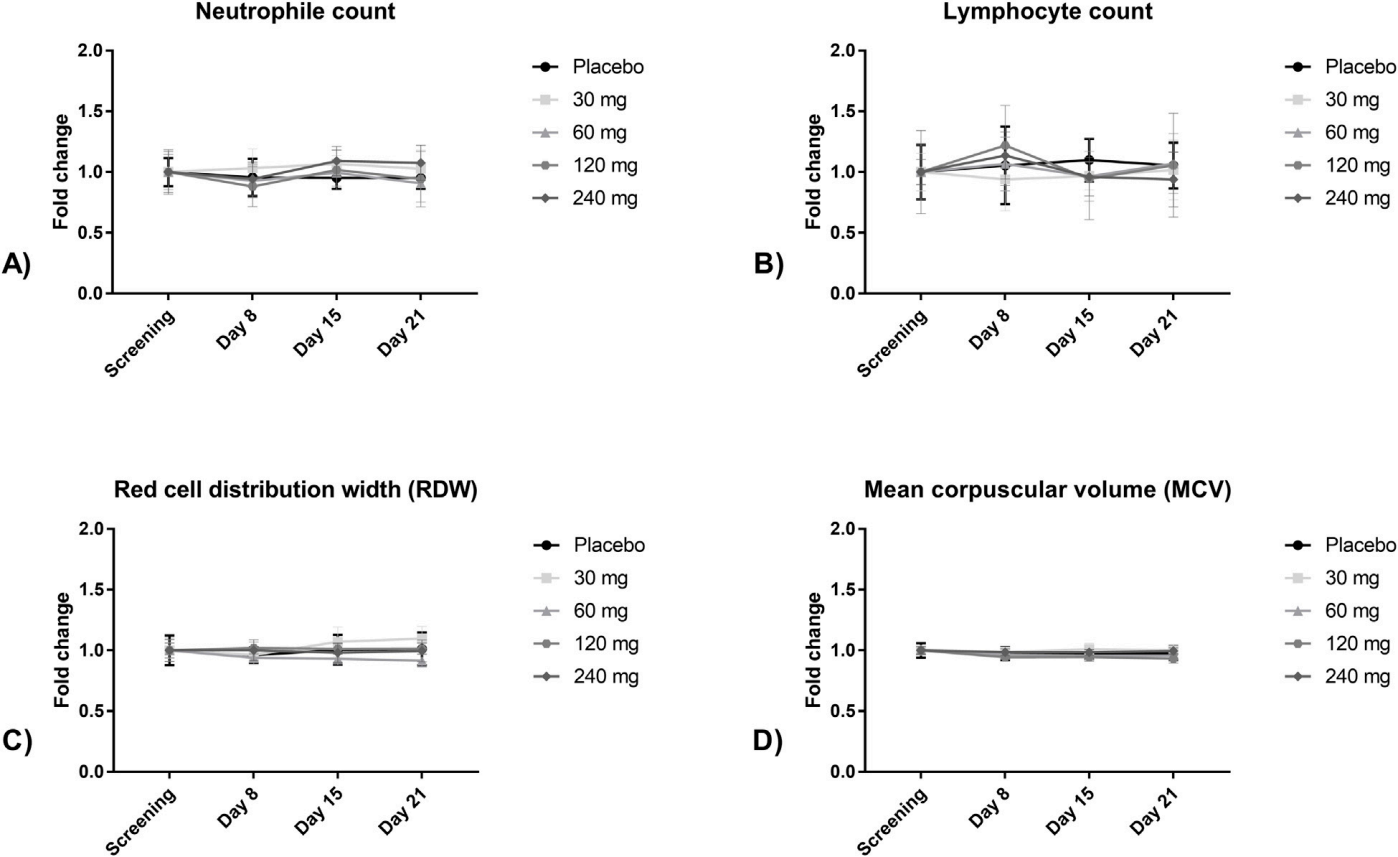
Fold change in mean **(A)** neutrophil count, **(B)** lymphocyte count, **(C)** red cell distribution width, and **(D)** mean corpuscular volume at screening, during, and after 14-day administration of CPL’116 b.i.d., n = 6 for each dose, n = 8 for placebo.

In the MAD part, AEs were reported in 19 of 32 participants (49%). The incidence of AE between the placebo and non-placebo groups was similar (50% vs 63%). The maximal severity of symptoms typically occurs shortly after the administration of the study product. The most frequently reported AE was headache [9 out of 43 (20.9%) AEs in 9 out of 32 (28.1%) participants]. No significant relationship between CPL’116 dose and the odds of any AE occurrence was identified [OR 1.005 (95% CI 1.000–1.012); p = 0.067]. Moderate AEs tended to be more frequently reported in higher-dose cohorts: three AEs (8%) in the 30 mg cohort, six AEs (16%) in the 60 mg cohort, eight AEs (22%) in the 120 mg cohort, and 20 AEs (54%) in the 240 mg cohort). Notably, only two AEs were deemed possibly related to the study product; however, both occurred in placebo participants. No clinically significant changes in neutrophil counts, red cell distribution width (RDW), or mean corpuscular volume (MCV) were observed after 14-day CPL’116 administration compared to placebo (Figure 2). Similarly, there were no clinically relevant differences in the lymphocyte levels between the placebo and active treatment groups. A decreased number of leukocytes was reported for two participants receiving 120 mg of CPL’116 and placebo. Hematocrit, platelet count, and creatine kinase levels were comparable between the placebo and CPL’116 groups (Supplementary Figures S1, S2). The mean aspartate transaminase (AST) and alanine transaminase (ALT) levels were comparable between the placebo and CPL’116 at doses in the range of 30–120 mg (Supplementary Figure S2). Mean AST and ALT values were slightly elevated during administration of CPL’116 at 240 mg dose, but none of the individual values beyond normal limits were classified as clinically relevant, and all values returned to the normal range 1 week after the last dose (Supplementary Table S1).

Vital signs, including sitting systolic and diastolic blood pressure (BP), heart rate (HR), body temperature (BT), and respiratory rate (RR), assessed across cohorts and measurement times, were within the normal range, except for isolated cases with values outside the normal range that were assessed as clinically non-significant.

### 3.3 Pharmacokinetics

**FIGURE 3:**
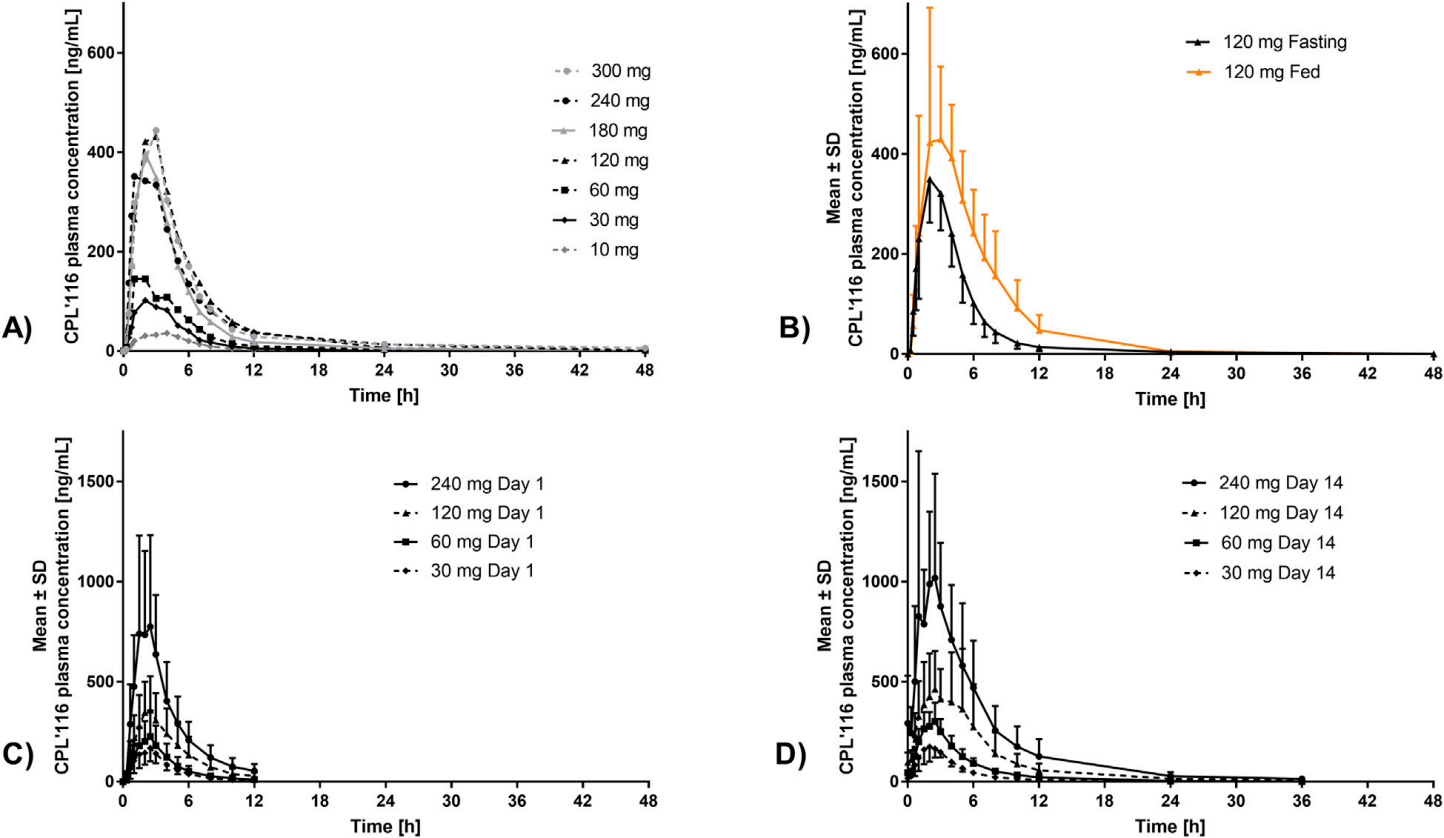
Mean plasma concentration-time plots in linear-linear scale, error bars indicate SD: **(A)** the SAD part, n = 3; **(B)** food effect part, n = 12; **(C)** Day 1 of the MAD part, n = 6; **(D)** Day 14 of MAD part, n = 6.

The mean CPL’116 plasma concentration vs time curves for the SAD, food effect, and MAD parts of the study (Days 1 and 14) are presented in Figure 3.

In the SAD part, the median t_max_ of CPL’116 in the plasma ranged from 2–3 h after administration and did not seem to be related to the dose (Supplementary Table S2). C_max_ and AUC_0–24h_ increased in each consecutive cohort, except for the 120 mg cohort due to a single outlier with a C_max_ of 719 ng/mL and AUC_0–24h_ of 4383 ng/mL∙h. The mean molar M3-to-CPL’116 ratio did not exceed 14% for C_max_ and 23% for AUC_0–24h_.

The geometric mean fed-to-fasting ratios for AUC_0–24h_, AUC_0-inf_, and C_max_ ranged from 139%–178%, indicating an increase in absorption by food intake (Supplementary Table S3). Food delayed the absorption of CPL’116 (median t_max_ difference 1 h, Wilcoxon test p-value: 0.095 - not statistically significantly different at α = 0.05).

In the MAD part, the mean C_max_, AUC_0–12h_, and AUC_0-inf_ were comparable between Days 1 and 14. Low to moderate variability in these parameters was observed between participants within the cohorts (Table 3). A proportional relationship between the above parameters and CPL’116 dose was observed in the range of 60–240 mg. The median t_max_ was not related to the dose. The mean t_1/2_ on Day 14 was significantly longer for higher doses (120 and 240 mg), possibly due to method sensitivity influencing the time of the last measurable concentration. Visual inspection of the concentrations observed in the pre-dose samples on each day suggested that a steady state was achieved on Day 2. The mean accumulation ratio for AUC_0–12h_ ranged from 1.3-1.6 (Day 8 vs Day 1) and from 1.2 to 1.8 (Day 14 vs Day 1). Inter-subject variability for C_max_ and AUC_0–12h_ between Day 8 and Day 14 was moderate.

**TABLE 3:** Plasma pharmacokinetic parameters of CPL’116 (in multiple ascending dose and food effect parts of study).

Dose (mg)	Day / Condition	n	AUC_0-12h_ (ng/mL∙h)	C_max_ (ng/mL)	t_max_ (h)	t_1/2_ (h)
Multiple ascending dose (MAD)						
30	Day 1	6	657 ± 188	184 ± 53	2.5 (1; 3)	-
30	Day 14	6	767 ± 207	183 ± 67	2.2 (1.5; 3)	6.1 ± 2.8
60	Day 1	6	904 ± 460	239 ± 122	2.5 (1.5; 2.5)	-
60	Day 14	6	1394 ± 334	306 ± 92	2.5 (1.5; 2.5)	5.9 ± 2.7
120	Day 1	6	1717 ± 759	398 ± 166	2.5 (1.5; 4)	-
120	Day 14	6	2901 ± 1375	581 ± 255	2.5 (2; 5)	9.3 ± 4.9
240	Day 1	6	3360 ± 1624	837 ± 450	2.5 (1.5; 3)	-
240	Day 14	6	5683 ± 2572	1211 ± 642	2 (1; 4)	8.0 ± 3.0
Food effect						
120	Fasting	12	1682 ± 435	361 ± 76	2 (2; 3)	9.0 ± 5.3
120	Fed	12	2975 ± 759	521 ± 194	3 (2; 5)	3.9 ± 0.7

Data are presented as mean and standard deviation, except for median (min; max) for t_max_. Note: sampling to 12 h was not long enough to properly characterize t_1/2._ Abbreviations: AUC_0-12h_, area under the plasma concentration-time curve up to 12 h (AUC in the dosing interval); C_max_, maximum observed plasma concentration; t_1/2_, terminal elimination half-life; t_max_, time of maximum observed plasma concentration.

### 3.4 Exploratory pharmacodynamics

**FIGURE 4:**
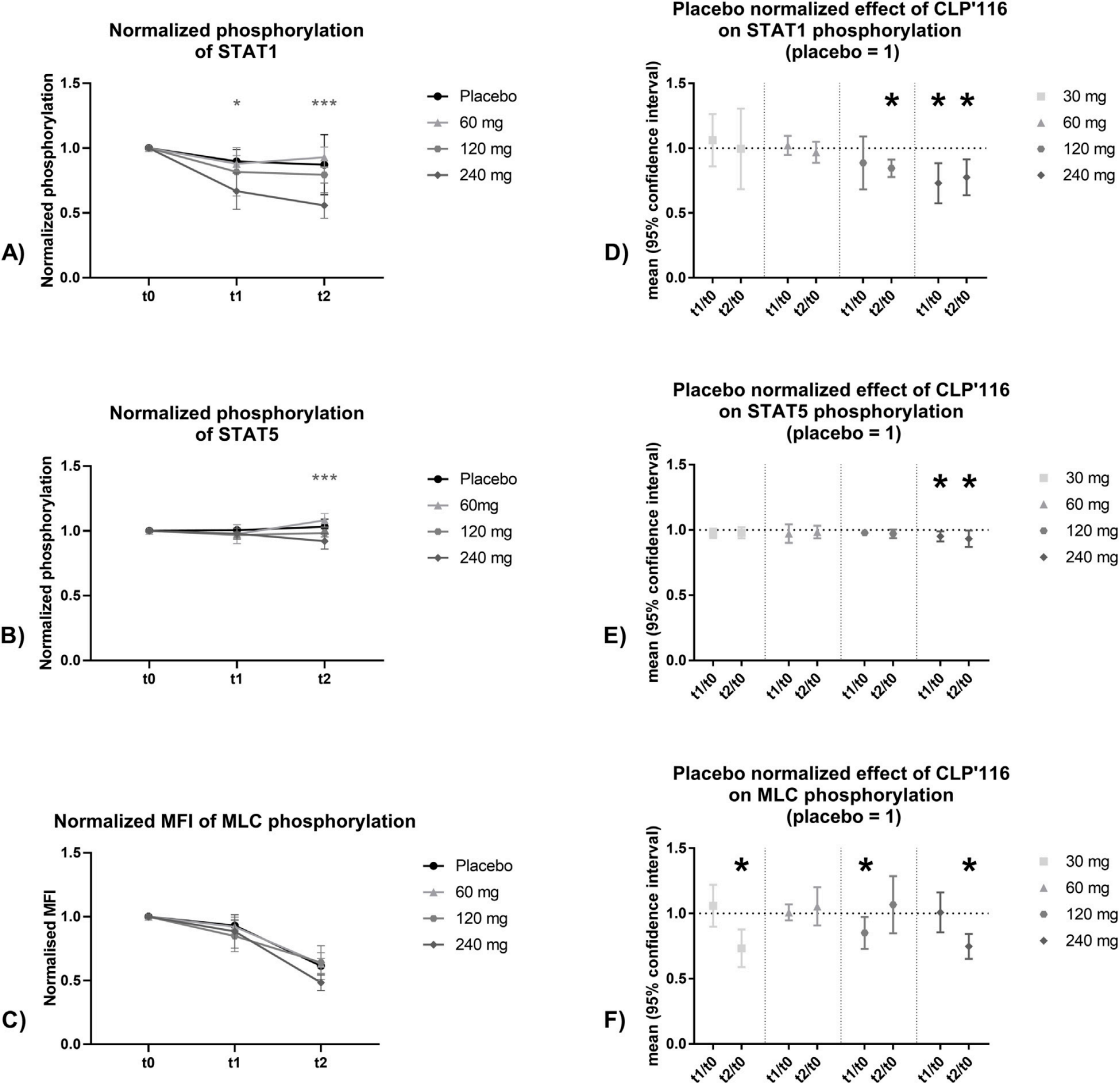
Results of exploratory PD study: t0-normalized median fluorescence intensity (MFI) of phosphorylation over time for STAT1 **(A)**, STAT5 **(B)**, and MLC **(C)**; placebo-normalized effect of CPL’116 on phosphorylation of STAT1 **(D)**, STAT5 **(E)**, and MLC **(F)**. Time points: t0 is pre-dose on Day 1; t1 is 2 h after administration on Day 1; t2 is 2 h after administration on Day 14.

The influence of multiple administrations of CPL’116 on the phosphorylation of STAT1, STAT5, and MLC proteins in whole blood is shown in Figure 4. To better understand the PD of CPL’116, two approaches to data analysis were applied: (1) normalization against pre-dose was conducted to eliminate between-subject variability and (2) after normalization against pre-dose, additional normalization against placebo was conducted to eliminate between-day variability.

After normalization against the pre-dose, for 240 mg b.i.d., *post hoc* Šidák multiple comparison test showed a significant decrease in STAT1 phosphorylation on Day 1 (−33% for CPL’116% vs −10% for placebo) and Day 14 (−44% for CPL’116% vs −13% for placebo) and in STAT5 phosphorylation on Day 14 (−8% for CPL’116 vs +3% for placebo). The decrease in MLC phosphorylation was not statistically significant, although the results suggest a potential trend at a significance level of 0.1. Given this assumption, the results for the 240 mg b.i.d. were −12% for CPL’116% vs −7% for placebo on Day 1% and -52% for CPL’116% vs −39% for placebo on Day 14.

After normalization against the pre-dose and placebo, the effect of CPL’116 on phosphorylation (Figures 4D–F) was significant for the following:

• STAT1 after 120 mg b.i.d. on Day 14 and 240 mg b.i.d. on Days 1 and 14
• STAT5 after 240 mg b.i.d. on Days 1 and 14
• MLC after 30 mg b.i.d. on Day 14, 120 mg b.i.d. on Day 1, and 240 mg b.i.d. on Day 14.

## 4 Discussion

The first-in-human study of CPL’116 was successfully completed, demonstrating that the compound was safe and well tolerated across all administered doses. No DLTs were observed following single or multiple doses, and no deaths or serious AEs occurred throughout the study.

Anticipated short-term side effects typically observed with JAK inhibition include diarrhea, nausea, headache, dyspepsia, and gastrointestinal discomfort. Clinical trials of tofacitinib (Papp et al., 2012; Vyas et al., 2013; Krishnaswami et al., 2015; Ma et al., 2024), baricitinib (Shi et al., 2014; Papp et al., 2016), filgotinib (Namour et al., 2015; Vanhoutte et al., 2017; Westhovens et al., 2025) and upadacitinib (Fleischmann et al., 2024; Vollenhoven et al., 2024; Wu Y et al., 2023) have highlighted the need for close monitoring of laboratory results due to potential dose-related cytopenias (e.g., anemia, neutropenia, thrombocytopenia, lymphopenia), elevated liver enzymes, disturbances in the lipid profile (increased HDL, LDL and total cholesterol levels) and decreases in hemoglobin levels. Adverse effects of ROCK inhibitors, as observed in clinical trials of fasudil (Wolff et al., 2024) and belumosudil (Schueller et al., 2024; Przepiorka et al., 2022) typically include hypo- or hypertension, headache, flushing, fatigue, and gastrointestinal discomfort. These side effects are generally mild; however, monitoring is necessary, especially for hypotension and hepatotoxicity, during the early stages of treatment.

CPL’116 did not demonstrate any AEs that were typically associated with JAK or ROCK inhibitors. Importantly, no unforeseen AEs emerged from the combined inhibition of the JAK and ROCK pathways in this early stage trial. We did not identify any safety concerns based on physical examination, occurrence of opportunistic infections, or vital signs. Although some individual cases of abnormalities in laboratory parameters were noted, they were not clinically relevant. We closely examined several parameters of interest to better assess the potential risks of CPL’116 (Figure 2). Over a 14-day period of b.i.d. CPL’116 administration, neutrophil and lymphocyte counts remained stable compared to those in the placebo group. Additionally, no changes in the red cell distribution width or mean corpuscular volume were observed. No clinically relevant increase in ALT or AST values was observed; however, a reversible increase in mean values during administration of the highest dose indicated that these parameters should be closely monitored for more than 14 days in future clinical studies. It should be noted that in the Phase II study [in publishing] of 106 patients with rheumatoid arthritis, no significant increase in ALT or AST levels after 3 months of CPL’116 administration was observed. The only commonly observed AE was headache, which occurred in 28% of participants who received multiple doses of the study drug. These results support the favorable safety profile of the investigational compound and provide a strong foundation for its further clinical development.

The sampling schedule and sensitivity of the bioanalytical method enabled proper calculation of PK parameters. The PK data were consistent across all parts of the study. The results indicated a less-than-proportional increase in the rate and extent of absorption in the dosing range of 10–300 mg, and dose linearity was observed for the selected parameters in limited dosing ranges. Food increased the rate and extent of absorption (C_max_ and AUCs were significantly greater in fed than in fasting conditions) at 120 mg; however, it is not yet known whether this is clinically relevant. A steady state may be achieved as early as Day 2, and CPL’116 does not accumulate to a considerable extent. Depending on dose and fasting/fed condition, mean terminal elimination half-life for CPL’116 ranged from 3.9–9.3 h (Table 3) and was comparable for other JAK inhibitors: 5.8–7.4 h (Zhao et al., 2020) or 8.5 h (Shi et al., 2014) for baricitinib, 5.9–14.5 h for upadacitinib (Mohamed et al., 2016), and 14–17 h for KL130008 (Li et al., 2022). Only tofacitinib had a shorter mean half-life of 3.2 h for immediate formulation and 5.9 h for extended-release formulation (Lamba et al., 2016). The ROCK inhibitor belumosudil has a comparable mean half-life of 7.5–10.9 h (Schueller et al., 2024), as does hydroxyfasudil, an active metabolite of the ROCK inhibitor fasudil, with a mean half-life of 5.5–5.7 h (Wolff et al., 2024). The half-life of CPL’116 supports twice-daily dosing and indicates low potential for accumulation, which contributes to the safety of this drug candidate.

Results from an exploratory PD study revealed a significant decrease in the phosphorylation of JAK downstream proteins (STAT1 and STAT5), as well as a trend toward decreased phosphorylation of the ROCK downstream target (MLC) in response to CPL’116 administration. These data represent the first indication of CPL’116’s ability to affect both JAK and ROCK pathways in humans.

The main limitations of this clinical trial were the small sample size, limited duration of multiple dosing, homogenous population consisting of healthy White subjects, and lack of observation of DLT. Most of these limitations are related to the first-in-human standard study design; for ethical reasons, both the number of subjects and the duration of treatment were limited. Inappropriate extrapolation and overinterpretation of outcomes should be avoided.

## 5 Conclusion

CPL’116, a novel dual inhibitor of JAK/ROCK, displayed a favorable safety profile in healthy participants and pharmacokinetic characteristics, supporting a twice-daily dosage. The results of our study provide a solid foundation for future clinical investigation. The dual mechanism of action of CPL’116 is particularly promising as it offers the potential to enhance the efficacy of the treatment of diseases characterized by both inflammatory and fibrotic changes, such as idiopathic pulmonary fibrosis, renal fibrosis, and interstitial lung disease associated with rheumatoid arthritis or systemic lupus erythematosus. Notably, a Phase II study in rheumatoid arthritis patients has since been completed successfully, demonstrating the significant efficacy and safety of CPL’116 [in publishing]. In future research, we will see a compelling prospect in mitigating cardiovascular comorbidities associated with autoimmune diseases, as well as safety concerns linked to JAK inhibition, by incorporating the cardioprotective properties of ROCK inhibition. However, this potential benefit can only be fully explored in large-scale studies conducted in later phases of clinical development.

## Data Availability

According to the sponsor’s data-sharing policy the datasets presented in this article are available from the sponsor upon reasonable request. Requests to access the datasets should be directed to marta.maciejak@celonpharma.com.
