# Supplementary material for: First-in-human study of CPL’116 – a dual JAK/ROCK inhibitor – in healthy subjects
Source: Front Pharmacol. 2025 Apr 1;16:1583723. doi: 10.3389/fphar.2025.1583723 (PMC11996792; doi:10.3389/fphar.2025.1583723)
Supplement: Supplementary file 1 [file DataSheet1.docx]

Supplementary Material

# Supplementary Data

## Bioanalytical method

Bioanalysis was performed at a GLP-certified laboratory of Celon Pharma S.A. (Kazuń Nowy, Poland) using liquid chromatography coupled to tandem mass spectrometry (1290 Infinity II, Agilent Technologies, coupled to QTrap 6500+; Sciex). CPL’116 and its M3 metabolite concentrations in human plasma were determined simultaneously using a Zorbax SB-C18 50 *×* 2.1 mm *×* 1.7 mm (Agilent Technologies) chromatographic column maintained at 50°C. Mobile phases A (1 mM aqueous NH_4_F) and B (mixture of methanol and water 95:5, v/v with addition of 0.1% acetic acid 0.1%) were used at a flow rate of 0.4 mL/min for gradient elution with %B ranging from 45% to 99%. Positive electrospray ionization was used to monitor CPL’116 at 499.2>215.2 m/z and its isotope labeled internal standard (d_3_-CPL’116) at 502.1>215.2 m/z, metabolite M3 at 513.3>282.4 m/z and its isotope labeled internal standard (d_6_-M3) at 519.2>133.2 m/z. The method was validated in the range
of 2-500 ng/mL for CPL’116 and 1-250 ng/mL for M3. Samples were prepared for analysis by protein precipitation using acetonitrile. All validation parameters met the acceptance criteria.

## Analysis of JAK activity by measurement of STAT1 and STAT5 phosphorylation

For STAT phosphorylation (STAT1 and STAT5), whole human blood was stimulated with recombinant cytokines (IL-6 and IL-15). After processing (red blood cell lysis, fixation, and permeabilization), samples were stained with fluorescently labelled antibodies specific to phosphorylated STAT1 and phosphorylated STAT5. STAT phosphorylation was determined based on the fluorescence intensity measured by fluorescence-activated cell sorting (FACS). The influence of the test compound on the phosphorylation status of signaling molecules was determined by comparing the percentage of cells positive for STAT staining in blood samples obtained before and after test compound administration. Samples were run in three technical replicates (n=3).

## Analysis of ROCK activity by measurement of MLC phosphorylation

For MLC phosphorylation, whole human blood was stained with a fixable viability dye, processed (red blood cell lysis, fixation, and permeabilization), samples were stained with fluorescently labelled antibodies specific to phosphorylated MLC. MLC phosphorylation was determined based
on the Median Fluorescence Intensity (MFI) measured by FACS. The influence of the test compound on the phosphorylation status of signaling molecules was determined by comparing the MFI in blood samples obtained before and after test compound administration. Samples were run in three technical replicates (n=3).

# Supplementary Figures and Tables

## Supplementary Tables

**Supplementary Table S1**. Categorization of AST and ALT values against normal range, including assessment of clinical significance for values beyond normal range at screening, during, and after
14-day administration of CPL’116 b.i.d.

| **Parameter** | **Day** | **Normal range** | **Placebo** | **30 mg** | **60 mg** | **120 mg** | **240 mg** |
| --- | --- | --- | --- | --- | --- | --- | --- |
| AST | Screening | within | 8 (100.0%) | 6 (100.0%) | 6 (100.0%) | 6 (100.0%) | 5 (83.3%) |
|  |  | beyond - clinically  not relevant | 0 (0.0%) | 0 (0.0%) | 0 (0.0%) | 0 (0.0%) | 1 (16.7%) |
|  |  | beyond - clinically relevant | 0 (0.0%) | 0 (0.0%) | 0 (0.0%) | 0 (0.0%) | 0 (0.0%) |
|  | Day 8 | within | 8 (100.0%) | 6 (100.0%) | 6 (100.0%) | 6 (100.0%) | 5 (83.3%) |
|  |  | beyond - clinically  not relevant | 0 (0.0%) | 0 (0.0%) | 0 (0.0%) | 0 (0.0%) | 1 (16.7%) |
|  |  | beyond - clinically relevant | 0 (0.0%) | 0 (0.0%) | 0 (0.0%) | 0 (0.0%) | 0 (0.0%) |
|  | Day 15 | within | 8 (100.0%) | 6 (100.0%) | 6 (100.0%) | 6 (100.0%) | 4 (66.7%) |
|  |  | beyond - clinically  not relevant | 0 (0.0%) | 0 (0.0%) | 0 (0.0%) | 0 (0.0%) | 2 (33.3%) |
|  |  | beyond - clinically relevant | 0 (0.0%) | 0 (0.0%) | 0 (0.0%) | 0 (0.0%) | 0 (0.0%) |
|  | Day 21 | within | 7 (87.5%) | 6 (100.0%) | 6 (100.0%) | 6 (100.0%) | 6 (100.0%) |
|  |  | beyond - clinically  not relevant | 1 (12.5%) | 0 (0.0%) | 0 (0.0%) | 0 (0.0%) | 0 (0.0%) |
|  |  | beyond - clinically relevant | 0 (0.0%) | 0 (0.0%) | 0 (0.0%) | 0 (0.0%) | 0 (0.0%) |
| ALT | Screening | within | 8 (100.0%) | 6 (100.0%) | 5 (83.3%) | 6 (100.0%) | 6 (100.0%) |
|  |  | beyond - clinically  not relevant | 0 (0.0%) | 0 (0.0%) | 1 (16.7%) | 0 (0.0%) | 0 (0.0%) |
|  |  | beyond - clinically relevant | 0 (0.0%) | 0 (0.0%) | 0 (0.0%) | 0 (0.0%) | 0 (0.0%) |
|  | Day 8 | within | 8 (100.0%) | 6 (100.0%) | 6 (100.0%) | 6 (100.0%) | 4 (66.7%) |
|  |  | beyond - clinically  not relevant | 0 (0.0%) | 0 (0.0%) | 0 (0.0%) | 0 (0.0%) | 2 (33.3%) |
|  |  | beyond - clinically relevant | 0 (0.0%) | 0 (0.0%) | 0 (0.0%) | 0 (0.0%) | 0 (0.0%) |
|  | Day 15 | within | 7 (87.5%) | 6 (100.0%) | 6 (100.0%) | 6 (100.0%) | 4 (66.7%) |
|  |  | beyond - clinically  not relevant | 1 (12.5%) | 0 (0.0%) | 0 (0.0%) | 0 (0.0%) | 2 (33.3%) |
|  |  | beyond - clinically relevant | 0 (0.0%) | 0 (0.0%) | 0 (0.0%) | 0 (0.0%) | 0 (0.0%) |
|  | Day 21 | within | 8 (100.0%) | 6 (100.0%) | 6 (100.0%) | 6 (100.0%) | 6 (100.0%) |
|  |  | beyond - clinically  not relevant | 0 (0.0%) | 0 (0.0%) | 0 (0.0%) | 0 (0.0%) | 0 (0.0%) |
|  |  | beyond - clinically relevant | 0 (0.0%) | 0 (0.0%) | 0 (0.0%) | 0 (0.0%) | 0 (0.0%) |

**Supplementary Table S2**. CPL’116 plasma pharmacokinetic parameters in the SAD study under fasting conditions (n = 3).

| **Dose  (mg)** | **AUC_0-24h_  (ng/mL∙h)** | **C_max_  (ng/mL)** | **t_max_  (h)** | **t_1/2_  (h)** |
| --- | --- | --- | --- | --- |
| 10 | 221 ± 159 | 41 ± 22 | 2 (2; 4) | 2.3 ± 0.5 |
| 30 | 537 ± 273 | 117 ± 41 | 2 (1; 3) | 2.7 ± 0.9 |
| 60 | 832 ± 334 | 190 ± 97 | 2 (1; 4) | 10.5 ± 3.8 |
| 120 | 2521 ± 1663 | 461 ± 240 | 3 (2; 3) | 4.9 ± 3.1 |
| 180 | 1935 ± 204 | 397 ± 67 | 2 (2; 2) | 8.1 ± 4.1 |
| 240 | 2220 ± 914 | 419 ± 30 | 2 (1; 3) | 8.8 ± 2.6 |
| 300 | 2382 ± 1055 | 488 ± 207 | 2 (1; 3) | 15.4 ± 7.6 |

Data are presented as mean and standard deviation, except for median (min; max) for t_max_. Abbreviations: AUC_0-24h_,
area under the plasma concentration-time curve up to 24 h; C_max_, maximum observed plasma concentration; SAD,
single ascending dose; t_1/2_, terminal elimination half-life; t_max_, time of maximum observed plasma concentration.

**Supplementary Table S3**. Influence of food on the rate and extent of CPL’116 absorption (food effect cohort, dose of 120 mg, n = 12).

| **PK parameter** | **Fed** | **Fasting** | **Fed/Fasting GMR (90% CI)** |
| --- | --- | --- | --- |
| AUC_0-24h_ (ng/mL∙h) | 2887 | 1626 | 178% (149-212%) |
| AUC_0-inf_ (ng/mL∙h) | 2918 | 1695 | 172% (144-205%) |
| C_max_ (ng/mL) | 492 | 353 | 139% (113-173%) |

Data in columns Fed and Fasting presented as the geometric mean. Abbreviations: AUC_0-24h_, area under the plasma concentration-time curve up to 24 h; AUC_0-inf_, area under the plasma concentration-time curve extrapolated to infinity; C_max_, maximum observed plasma concentration; CI, confidence interval; GMR, geometric mean ratio

## Supplementary Figures

**Supplementary Figure S1**. Fold of change in mean (± standard deviation) hematocrit (HCT)
and platelet count (PLT) at screening, during, and after 14-day administration of CPL’116 b.i.d.

**Supplementary Figure S2**. Fold of change in mean (± standard deviation) aspartate transaminase (AST), alanine transaminase (ALT), and creatine kinase (CK) at screening, during, and after 14-day administration of CPL’116 b.i.d.
